# Contrasting Effects of Mutualistic Ants (*Solenopsis invicta)* and Predatory Ladybugs on the Proportion of Dark Green Morphs of Cotton Aphids

**DOI:** 10.3390/insects16030271

**Published:** 2025-03-04

**Authors:** Yao Chen, Hejun Cui, Tian Xu, Li Chen

**Affiliations:** 1 College of Life Sciences, Hebei Basic Science Center for Biotic Interactions, Hebei University, Baoding 071002, China; chen1050662425@163.com (Y.C.); 15128057421@163.com (H.C.); 2 Co-Innovation Center for the Sustainable Forestry in Southern China, Nanjing Forestry University, Nanjing 210037, China

**Keywords:** ant–aphid interaction, predator, invasive species, polymorphism, spatial variation, within-plant distribution, body color

## Abstract

Cotton aphids, *Aphis gossypii*, are an important pest worldwide and have evolved mutualistic relationships with the invasive fire ant *Solenopsis invicta*. Their body color varies from pale yellow to dark green, with an increase in body size and fecundity. The body color composition in a cotton aphid colony can be influenced by biotic interactions with mutualistic ants and predatory ladybugs. However, since the distribution of nutrients varies across host plant organs, there may exist special effects of biotic interactions on the body color composition of the aphids on different plant parts. In the present study, we found that, under constant laboratory conditions, the proportions of dark green morphs varied among the cotton aphids distributed on different parts of a cotton seedling, with significantly higher proportions on the stems, petioles, and sprouts (SPSs) than on leaves. The presence of mutualistic fire ants significantly increased the proportion of dark green morphs in the cotton aphid colony, but with a reduction in aphid body size, compared to the untended individuals. In contrast, the introduction of a predatory seven-spotted ladybug, *Coccinella septempunctata*, dramatically decreased the proportion of dark green morphs on SPSs, but not on leaves, leading to a reduction in the proportion of the whole colony. These results illustrate a spatial variation in the proportions of dark green morphs on host plants in cotton aphids, which may be an adaptive strategy used by the aphids to gain benefits and/or minimize costs in the interactions with mutualistic ants and predatory ladybugs.

## Simple Summary

The cotton aphid *Aphis gossypii* has polymorphisms in body color ranging from pale yellow to dark green. Aphid body color may undergo different adaptive changes in two habitats of mutualistic ants and predatory ladybugs. In this study, we compared the effects of mutualistic ants or predatory ladybugs on the body color of aphids feeding on different parts of cotton seedlings. We found that cotton aphids had a significantly higher proportion of dark green morphs on stems, petioles, and sprouts (SPSs) than on leaves. The presence of mutualistic ants increased the proportion of dark green morphs distributed on SPSs and leaves, but aphid body size was significantly reduced compared to the untended individuals. In contrast, the presence of predatory ladybugs *Coccinella septempunctata* decreased the proportion of dark green morphs distributed on the SPSs but did not affect the proportion on the leaves. These results suggest that spatial variation in the proportion of dark green morphs of cotton aphids on host plants is an adaptation to protect from mutualistic ants and risk from predatory ladybugs.

## 1. Introduction

Aphids are a group of sap-sucking insects, and many of them are important pests because of their large population sizes and ability to carry and transmit plant viruses. They are generally small insects with soft bodies. To defend against natural enemies such as ladybugs (Coleoptera: Coccinellidae), lacewings (Neuroptera: Chrysopidae), gophers (Diptera: Syrphidae), and parasitic wasps (Hymenoptera), aphids have evolved some defensive adaptations, i.e., siphunculi and their secretions, dense wax cover, drop-off, and thanatosis [1,2,3,4]. Some aphid species have also evolved alternative defense strategies by establishing food-based mutualistic relationships with ants [5]. They excrete excess sugars gained from host plants, along with water, as honeydew, which is a tasty meal for the mutualistic ants. The ants protect the aphids from their natural enemies to keep the honeydew resources [6,7,8].

Many aphids have polymorphisms in body color. For example, the pea aphid *Acyrthosiphon pisum* has two body colors, green and red, and the peach aphid *Myzus persicae* has three body colors, yellow, green, and red. The polymorphisms of aphids can occur within the same population or shift rapidly between generations [9]. The body color of aphids is affected by many factors, such as temperature, host species, light, symbiotic bacteria, and host plant quality [9]. Natural enemies and mutualistic ants also influence the body color composition in aphid populations. The seven-spotted ladybug *Coccinella septempunctata* prefers to prey on the red morphs in pea aphids, while the green morphs are more parasitized by parasitic wasps [10]. The dark green morph of cotton aphid *Aphis gossypii* is more susceptible to parasitic wasps than lighter-body-color morphs [11]. The presence of the mutualistic ant *Lasius japonicus* can change the proportions of green and red morphs in the aphid *Macrosiphoniella yomogicola* populations [12].

As phytophagous insects, the performances of aphids are closely related to the conditions of host plants. On plants with higher levels of nutrients, aphids produce offspring that are larger, grow faster, and are more fecund [13,14]. Nutrients in plant sap are thought to determine the food quality for aphids, and increased levels of these nutrients can promote aphid growth and reproduction [13,15]. However, the distribution of plant nutrients across plant organs is not uniform [16,17]. Thus, feeding on different parts of a plant can directly affect aphid performance. For example, populations of the aphid *Macrosiphoniella tanacetaria* feeding on the stems of *Tanacetum vulgare* grow faster than their counterparts feeding on the leaves [18]. Furthermore, host plant condition can also affect the ants through the aphid-produced honeydew, a bottom-up effect [19]. For example, defensive substances in plant sap are taken up by aphids and then excreted out through honeydew. Honeydew containing these substances has an avoidance effect on ants, which in turn attenuates their mutualistic relationship with the aphids [20,21]. To gain more high-quality honeydew, some ants actively move tended aphids to feed on young plant parts or plants with higher nutritional value [22,23].

The red imported fire ant (RIFA), *Solenopsis invicta*, is a highly destructive invasive species and has established extensive mutualistic relationships with cotton aphids in the southern United States since its invasion of North America [24,25]. The RIFA suppresses the populations of natural enemies in cotton fields, leading to the outbreaks of some major pests such as the cotton aphid [26,27]. Cotton aphids have a wide range of body colors, including yellow, light green, and dark green. Individuals with a certain body color can produce offspring of various body colors [28]. Yellow morphs are the smallest in body size, with the slowest growth rate and the lowest fecundity, while dark green morphs are the largest, with the fastest growth rate and the highest fecundity [29,30]. The body color of cotton aphids can be influenced by temperature, host species, host quality, and biotic interactions [11]. Yellow morphs are mainly produced at temperatures above 25 °C [30], and darker green morphs are observed on the cotton plants with higher nitrogen content [13]. The body color composition of a cotton aphid colony can also be influenced by interactions with mutualistic ants and natural enemies. When tended by the mutualistic Argentine ant *Linepithema humile*, the cotton aphids produce more light green morphs in the offspring [11], whereas the presence of the predatory ladybug *Hippodamia convergens* leads to more winged morph in the offspring that further produce mainly yellow morphs [28].

We have previously observed opposite effects of mutualistic RIFAs and predatory ladybugs on the distribution pattern of cotton aphids on cotton seedlings, with more individuals distributed on the stems, petioles, and sprouts in the presence of ants but more on the leaves in the presence of ladybugs (unpublished data). Since food quality is an important factor influencing the variation in aphid body color and varies across different parts of host plants [9], we hypothesized that the body color compositions varied among the cotton aphid morphs distributed on different plant parts, which can be impacted by the biotic interactions and may have adaptive significance. To test this hypothesis, we investigated the changes in the body color compositions, in particular the dark green morphs, of cotton aphids distributed on different parts of a cotton seedling in the presence of mutualistic RIFAs or predatory seven-spotted ladybugs compared to the colonies without any interspecific interactions under constant laboratory conditions.

## 2. Materials and Methods

### 2.1. Insects and Plants

*Solenopsis invicta* colonies were collected from the campus of South China Agricultural University (Guangzhou, China), brought back to the laboratory (26 °C, 70% RH, and L14:D10 photoperiod), and fed with 10% sugar water and freeze-killed crickets. Cotton aphids were collected from the Langfang Experimental Base of the Institute of Plant Protection, Chinese Academy of Agricultural Sciences (Langfang, China). Cotton aphids were raised in an incubator (RXZ-500, Jiangnan Instrument Factory, Ningbo, China; 25 °C, 70% RH, and L16:D8 photoperiod) with cotton seedlings (*Gossypium hirsutum* L., cultivar: Xinluzao 41) in the laboratory. This cultivar of cotton was also used in the subsequent experiments. Seven-spotted ladybugs, *Coccinella septempunctata*, were collected from oilseed rape plants in Shuyang County, Suqian, China, reared in the laboratory under ambient temperature (~23 °C), and fed with cotton aphids.

### 2.2. Effects of Ant-Tending and Plant Parts on Body Color and Size of Cotton Aphids

**Figure 1:**
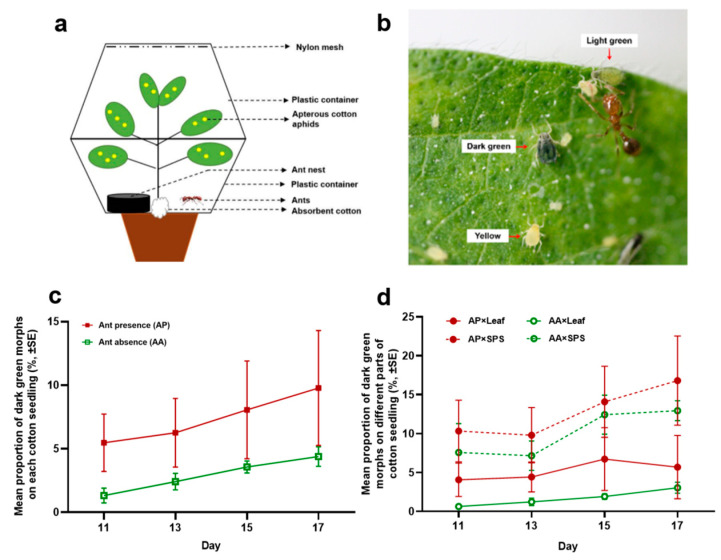
Effects of *S. invicta* attendance and plant part on the body color of cotton aphids. (**a**) The experimental set-up. (**b**) Three typical body colors of cotton aphids, including yellow, dark green, and light green, the latter an intermediate level of greenness with a gradual change between dark green and yellow. The proportion (**c**) of dark green morphs on a seedling on days 11, 13, 15, and 17, in the presence (in red) or absence (in green) of ants. The proportion (**d**) of dark green morphs among the total number of aphids distributed on different parts of seedlings (leaf, solid line; SPS, dash line), in ant presence (AP, in red) or absence (AA, in green), on days 11, 13, 15, and 17.

The ant–aphid interaction systems were set up as shown in Figure 1a. By using a heat gun, a circular hole (2 cm diameter) was punched in the bottom center of a transparent plastic container [3.5 L; diameter: 21 cm (open-top) and 17.5 cm (bottom); height: 13 cm]. The container was then cut into two halves across the hole. The stem of a cotton seedling (~5 weeks old, with 5–6 fully expanded leaves) cultivated in a pot was wrapped with cotton wool (2–3 cm above the soil surface) and held by the two cut halves through the hole. The gap between the cotton wool and the container was sealed together with a hot melt glue gun. The inner wall of the container (mutualistic group and non-mutualistic group) was coated with PTFE suspension (Dongguan Mingde Plastic Co., Ltd., Dongguan, China) to prevent insects from escaping.

A total of 16 systems were prepared. Fifty apterous cotton aphids were gently transferred onto the leaves with a brush. In each of the eight systems, a sub-colony of polygynous *S. invicta* was introduced, consisting of 1 g of workers (~1000 individuals), one queen, and 20–30 larvae. A plastic Petri dish (6 cm diameter) was placed into the system as an artificial nest for ants. The bottom of the Petri dish was covered with a layer of plaster (~0.5 cm thickness) which was moisturized every other day after ants were introduced. The top of the Petri dish was covered with black tape to keep the nest dark. In the other eight systems, no ants were introduced, and these served as non-mutualistic controls. The systems in the same treatment group were kept in a climate incubator (25 °C, 60–70% RH, 16 L:8 D), and the seedlings were watered every 2–3 days. Nine days after the aphid colony had been established, dark green morphs were observed on each seedling (Figure 1b). Thus, the numbers of aphids in all morphs as well as the numbers of dark green morphs distributed on leaves (cotyledons and fully expanded leaves) and SPSs (stems, petioles, and sprouts) of each seedling were counted on days 11, 13, 15, and 17. In a few systems, the ants (including the queen) escaped out of the device or the seedling died on days 15 and 17, the data from which, thereby, were not included in the analysis.

On day 18, a total of eighteen adult aphids in dark green or yellow were collected from the leaves and SPSs, in both mutualistic and non-mutualistic systems, which were then killed at −20 °C. A Leica M205A microscope (Wetzlar, Germany) was used to take a picture of each aphid. The body lengths (front of head to cauda [13]) and head widths (distance between the two compound eyes) of these aphids were measured by using ImageJ 1.48v software (National Institutes of Health, Bethesda, MD, USA).

### 2.3. Effects of Predatory Ladybug on Body Color of Cotton Aphids

Fifty apterous cotton aphids (yellow morph, 4th instar) were evenly placed on the upper side of the leaves on each of sixteen cotton seedlings (~5 weeks old, with 5–6 fully expanded leaves) which were prepared in the systems as described above (Figure 1a). The seedlings were kept in two climate incubators (8 in each, 25 °C, 60–70% RH, 16 L:8 D). After the aphid colonies had grown without ant attendance for two weeks, the numbers of all aphids and dark green morphs on leaves and SPS of each seedling were counted (day 0). The seedlings were then randomly divided into two groups (8 each), which had similar numbers of aphids and proportions of dark green morphs on both leaves and SPSs. On day 0, an adult ladybug (1–2 weeks old, starved for 24 h) was introduced to each seedling of one group (ladybug presence, *n* = 8), and the colonies on the other seedlings continued to grow without predator attacks (ladybug absence, *n* = 8). The seedlings in the same treatment were kept in a climate incubator under the same environmental conditions as above. The numbers of all aphids and dark green morphs on the leaves and SPSs of each seedling were counted three times every other day (i.e., days 2, 4, and 6).

### 2.4. Statistical Analysis

A generalized linear model was used to analyze the effects of day, ant attendance (ant presence and ant absence), plant part (leaf and SPS), and their interactions (day × ant, day × plant part, ant × plant part, and day × ant × plant part) on the proportion of dark green morphs. This model was also used to analyze the effects of plant part (leaf and SPS), ant attendance (ant presence and ant absence), body color (dark green and yellow), and their interaction (body color × plant part, body color × ant, ant × plant part, and body color × plant part × ant) on the body length and head width of adult cotton aphids. A generalized linear model was used to analyze the effects of the predatory ladybug (ladybug presence and ladybug absence), plant part (leaf and SPS), and their interaction (ladybug × plant part) on the proportion of the dark green morphs. If any interaction effects were significant in the analyses, a post hoc pairwise comparison was carried out. The detailed scripts of the GLM can be found in Supplementary Materials. All statistical analyses in this study were performed by using SPSS 22.0 (SPSS, Armonk, NY, USA).

## 3. Results

### 3.1. Effects of Ant-Tending and Plant Parts on Body Color and Size of Cotton Aphids

#### 3.1.1. Body Color

The overall proportions of dark green morphs in the whole cotton aphid colony on a cotton seedling were significantly higher when tended by RIFAs (Figure 1c). The plant part and presence of mutualistic ants were two main factors affecting the proportions of dark green cotton aphids, with significantly higher proportions of dark green morphs on SPSs and in the presence of mutualistic ants. No significant difference was found among different observation days (Figure 1 and Table 1).

#### 3.1.2. Body Size

**Figure 2:**
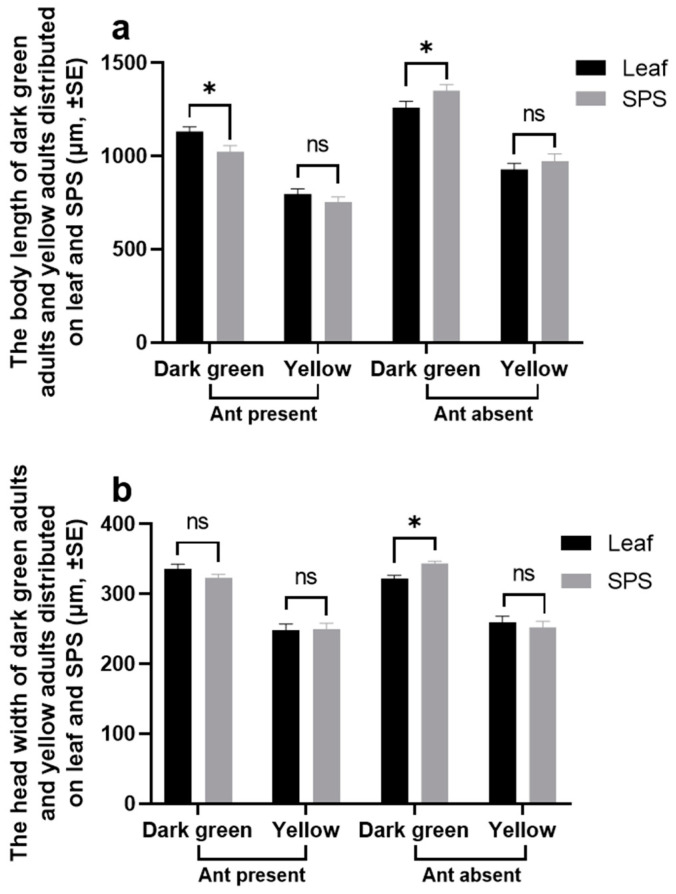
The body length (**a**) and head width (**b**) of dark green adults and yellow adults distributed on leaves and SPSs (stems, petioles, and sprouts) on day 18 (*n* = 18). Asterisks indicate statistically significant differences between treatments (* *p* < 0.05, LSD), ns = not significant.

The body lengths and head widths of dark green morphs on SPSs and leaves were both significantly larger than those of yellow morphs (Figure 2 and Table 2). The body lengths of dark green and yellow aphids in the ant-tended colonies were significantly smaller than those of non-ant-tended aphids, but there was no significant difference in the head widths (Figure 2 and Table 2). There was significant interaction between the two main effects (ant × plant part) on body length (Table 2; Wald’s chi-square = 10.696, *p* = 0.0011). The body lengths of aphids on leaves, in the presence of ants, were larger than those on SPSs, while, in the absence of ants, they were smaller than those on SPSs (LSD: ants presence, leaf vs. SPS, *p* = 0.0154; ants absence, leaf vs. SPS, *p* = 0.0277). The interaction effect was significant between the three main effects on head width (ant × plant part × body color) (Table 2; Wald’s chi-square = 5.158, *p* = 0.0231). The pairwise comparisons showed that, in the absence of ants, the dark green morphs distributed on SPSs had larger head widths than those on leaves (LSD: *p* = 0.0202); on SPSs, the head widths of dark green morphs in the absence of ants were larger than those in the presence of ants (LSD: *p* = 0.0350).

### 3.2. Effect of Predatory Ladybugs on Body Color of Cotton Aphids

**Figure 3:**
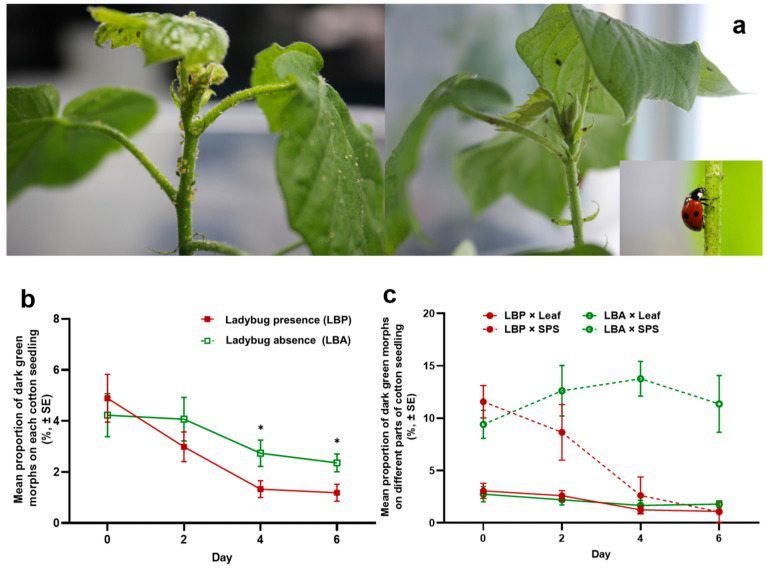
Effects of predatory ladybugs on the within-plant distribution and body color of cotton aphids. The distributions of cotton aphids on a cotton seedling, in the absence and presence (**a**) of seven spotted ladybugs, *Coccinella septempunctata*. (**b**) The proportions of dark green aphids on the whole seedling on days 0, 2, 4, and 6, in the presence (in red) or absence (in green) of ladybugs. (**c**) The proportions of dark green aphids on leaves (solid line) or SPSs (dash line) of cotton seedlings, in the presence (LBP, in red) or absence (LBA, in green) of ladybugs. Asterisks indicate statistically significant differences between treatments on the same day (* *p* < 0.05, LSD).

The presence of predatory ladybugs significantly affected the distribution of dark green morphs on cotton seedlings (Figure 3a). On the whole seedling, the proportions of dark green morphs in the colonies attacked by a ladybug were significantly smaller than those in the unattacked colonies on days 4 and 6 (Figure 3b and Table 3). On days 0 and 2, plant part was the only main factor affecting the proportion of dark green morphs, with significantly larger proportions on SPSs than on leaves. On day 4, both plant part and ladybug showed significant effects on the proportion of dark green morphs, with a significant interaction effect between them. On day 6, there was also a significant interaction effect. The presence of a ladybug did not have an effect on the proportion of dark green aphids distributed on leaves (LSD: ladybug presence vs. ladybug absence, day 2, *p* = 0.8695; day 4, *p* = 0.7949; day 6, *p* = 0.7208), but the proportion on SPSs quickly decreased (LSD: ladybug presence vs. ladybug absence, day 2, *p* = 0.1017; day 4, *p* < 0.0001; day 6, *p* < 0.0001), leading to an increase in the proportion of dark green aphids on leaves on days 2 and 4.

## 4. Discussion

Color polymorphisms are a common feature of many aphid species. Cotton aphids exhibit a wide variety of body colors, ranging from pale yellow to dark green. In the present study, we found that the proportion of dark green morphs of cotton aphids on stems, petioles, and sprouts (SPSs) was significantly higher compared to that on leaves under constant laboratory conditions. The presence of mutualistic fire ants increased the proportion of dark green morphs among the cotton aphids distributed on either leaves or SPSs, whereas the presence of predatory ladybugs led to an opposite effect on the proportion of dark green morphs, particularly on SPSs. However, significantly decreased body size was also found in the aphids, with the same body color, distributed either on SPSs or leaves that were tended by ants, suggesting the presence of a cost generated from the interactions with ants.

As phytophagous insects generally prefer to feed on more nutritious plant parts, the spatial distribution of nutrients in host plants can directly shape the within-plant distribution pattern of aphids [31,32]. In plants, the vigorously growing parts (e.g., upper parts of stems, newborn leaves and sprouts) may contain relatively higher levels of soluble nitrogenous substances due to high protein synthesis or degradation activities than mature leaves [16,17]. A previous study has reported that the elevated concentration of nitrogen in cotton plants can increase the proportion of dark green morphs in cotton aphid colonies [13], which may explain the higher proportion of dark green morphs on SPSs observed in this study. Moreover, our results showed that the body sizes of the cotton aphids distributed on SPSs were larger than those on leaves. These findings suggest better performance of cotton aphids on SPSs than on leaves.

In the present study, we found that the proportions of dark green morphs declined in the cotton aphid colony after a predatory ladybug was introduced, which was mainly due to a dramatic decrease in the dark green morph proportion on SPSs compared to that on leaves. Predatory ladybugs have been found to be able to evaluate the quality of prey based on the organs of the host plant to save time and energy [33]. We observed that ladybugs spent longer periods of time moving or staying on the upper part of cotton seedlings. Aphids may be able to sense ladybug activity and thus quickly move to the leaves or drop off from the plant to escape predation [34]. Predators are able to select more nutrient-dense foods to increase predation efficiency [35]. Seven-spotted ladybugs can recognize prey by exploiting color differences between the prey and the background [36]. Hence, seven-spotted ladybugs may be able to discriminate between dark green and yellow cotton aphid individuals by vision, with a potential preference for dark green morphs, and forage more frequently in the areas mainly occupied by dark green individuals. In addition to predatory natural enemies, parasitoid wasps are more likely to attack the dark green morphs in the cotton aphid colonies [28]. These findings imply that dark green morphs probably suffer greater attack by natural enemies than yellow morphs, and the reduction in the proportion of dark green morphs, particularly among the individuals on SPSs, may decrease the attacking risks from natural enemies in the absence of protective ants.

In most mutualistic relationships, members of different parties pool complementary abilities by exchanging services for their mutual benefit [37]. Although we did not observe the behavior of ants carrying aphids to specific parts of the plant during the experiment, the ants could use other means such as chemical signals to regulate the behavior of aphids. A recent study has found that perception of RIFA trail pheromones alters the behavior and reproductive performances of cotton aphids [38]. Thus, aphids that are attended by ants may have sensed the presence of ants, resulting in a change in the production of offspring in different body colors that differed from those of the control. The higher proportion of larger morphs in a cotton aphid colony may guarantee ants access to more honeydew resources, allowing them to reap more rewards from the mutualistic relationship.

The mutualistic associations between *S. invicta* colonies and honeydew-producing hemipterans in invaded areas are widespread [39]. The invasive fire ants monopolize carbohydrate sources provided by the hemipterans over native ant species [40,41]. Exploitation of honeydews by RIFAs is important in their invasion success [42]. The RIFA-tended cotton aphid colonies gain benefits from ant protection, having much larger population sizes than the untended colonies [43]. The increased proportions of dark green morphs in the RIFA-tended aphid colonies may also account for the rapid increase in colony size of cotton aphids, which, in return, facilitates outbreaks of invasive fire ants.

Aphids have been found to show some changes in behavior, life history, wing differentiation, and body structure in order to be more adapted to mutualistic ants [2,44,45]. However, the mutualism associated with ants can impose some negative effects on aphids, leading to smaller body size, slower individual development, and reduced reproductive capacity. These effects are thought to be attributed to the high demand for honeydew by the mutualistic ants and force the aphids to use more energy to secrete more honeydew or synthesize sugars preferred by the ants [46,47,48,49]. In the present study, we found that the cotton aphids in either yellow or dark green that were attended by ants were smaller in body size than their counterparts without ant-tending, suggesting a negative effect of ant attendance on cotton aphids. However, for aphids, the negative effects may be far less than the attrition caused by producing winged offspring that escape the danger [50]. Therefore, the results of the present study suggest that cotton aphids may be able to take advantage of ant protection as a favorable condition by distributing on nutrient-rich parts of plants, thereby producing larger, faster-growing, and more fecund phenotypes to satisfy the ants’ demand for honeydew and to compensate for the costs from mutualism.

In summary, the present study found that the proportion of dark green morphs in cotton aphids is closely related to their distribution sites on host plants, with a higher proportion on the nutritious parts, revealing a potential strategy for the aphids to actively change the colonial body color composition. By using this adaptive strategy, aphids can increase the proportion of dark green morphs that are larger and more fecund to gain more benefits and/or minimize the costs when the mutualistic ants are present, and decrease the proportion of dark green morphs that have greater attacking risks from natural enemies in the absence of mutualistic ants. These findings improve our understanding of the ecological implications of body color polymorphisms in aphids.

## Figures and Tables

**Table 1:** The effect of day, ant, and plant part on the proportion of dark green morphs (generalized linear modeling).

Source of Variation	*d.f.*	Wald’s Chi-Square	*p*
Intercept	1	122.912	<0.0001
Ant	1	5.423	0.0199
Plant part	1	34.933	<0.0001
Day	3	7.191	0.0660
Ant × plant part	1	0.089	0.7652
Plant part × day	3	2.016	0.5691
Ant × day	3	0.010	0.9997
Ant × day × plant part	3	0.311	0.9580

**Table 2:** The differences in the body lengths and head widths of the dark green and yellow adults distributed on different parts of seedlings, in the presence or absence of ants (generalized linear modeling).

Source of Variation	*d.f.*	Body Length		Head Width	
		Wald’s Chi-Square	*p*	Wald’s Chi-Square	*p*
Intercept	1	8985.049	<0.0001	14,566.087	<0.0001
Ant	1	86.911	<0.0001	1.208	0.2717
Plant part	1	0.025	0.8748	0.029	0.8643
Body color	1	230.292	<0.0001	266.645	<0.0001
Ant × plant part	1	10.696	0.0011	1.839	0.1751
Plant part × body color	1	0.038	0.8461	0.715	0.3979
Ant × body color	1	1.495	0.2214	0.26	0.6100
Ant × plant part × body color	1	1.690	0.1936	5.158	0.0231

**Table 3:** The difference between the number of aphids distributed on different parts of seedlings, in the presence or absence of predatory ladybugs on days 0, 2, 4, and 6 (generalized linear model, related to Figure 3b).

Day	Source of Variation	*d.f.*	Wald’s Chi-Square	*p*
0	Intercept	1	156.424	<0.0001
	Ladybug	1	1.341	0.2468
	Plant part	1	50.326	<0.0001
	Ladybug × plant part	1	0.752	0.3860
2	Intercept	1	57.860	<0.0001
	Ladybug	1	1.084	0.2978
	Plant part	1	23.101	<0.0001
	Ladybug × plant part	1	1.622	0.2028
4	Intercept	1	68.119	<0.0001
	Ladybug	1	24.563	<0.0001
	Plant part	1	33.260	<0.0001
	Ladybug × plant part	1	21.054	<0.0001
6	Intercept	1	30.830	<0.0001
	Ladybug	1	16.013	0.0001
	Plant part	1	11.970	0.0005
	Ladybug × plant part	1	12.223	0.0005

## Data Availability

Data are contained within the article.

## Supplementary Materials

The following supporting information can be downloaded at: https://www.mdpi.com/article/10.3390/insects16030271/s1, The scripts of Generalized Linear Model used in the present study.

## References

[1] Bilska A., Francikowski J., Wyglenda A., Masłowski A., Kaszyca N., Depa Ł. (2018). Aphids playing possum—Defensive or mutualistic response?. J. Insect Behav..

[2] Depa L., Kaszyca-Taszakowska N., Taszakowski A., Kanturski M. (2020). Ant-induced evolutionary patterns in aphids. Biol. Rev..

[3] Węgierek P., Malik K., Hutyra P., Depa Ł. (2025). What does the morphological diversity of siphunculi tell us about the evolution of aphids (Insecta, Hemiptera, Aphidoidea)?. Eur. Zool. J..

[4] Banks C.J. (1962). Effects of the ant *Lasius niger* (L.) on insects preying on small populations of *Aphis fabae* Scop. on bean plants. Ann. Appl. Biol..

[5] Stadler B., Dixon A.F.G. (2005). Ecology and evolution of aphid-ant interactions. Annu. Rev. Ecol. Evol. Syst..

[6] Way M.J. (1963). Mutualism between ants and honeydew-producing Homoptera. Annu. Rev. Entomol..

[7] Buckley R.C. (1987). Interactions involving plants, Homoptera, and ants. Annu. Rev. Ecol. Syst..

[8] Hölldobler B., Wilson E.O.B. (1990). The Ants.

[9] Tsuchida T. (2016). Molecular basis and ecological relevance of aphid body colors. Curr. Opin. Insect Sci..

[10] Losey J.E., Ives A.R., Harmon J., Ballantyne F., Brown C. (1997). A polymorphism maintained by opposite patterns of parasitism and predation. Nature.

[11] Mondor E.B., Rosenheim J.A., Addicott J.F. (2008). Mutualist-induced transgenerational polyphenisms in cotton aphid populations. Funct. Ecol..

[12] Watanabe S., Murakami T., Yoshimura J., Hasegawa E. (2016). Color polymorphism in an aphid is maintained by attending ants. Sci. Adv..

[13] Nevo E., Coll M. (2001). Effect of nitrogen fertilization on *Aphis gossypii* (Homoptera: Aphididae): Variation in size, color, and reproduction. J. Econ. Entomol..

[14] Honěk A., Martinková Z. (2002). Factors of between- and within-plant distribution of *Metopolophium dirhodum* (Hom., Aphididae) on small grain cereals. J. Appl. Entomol..

[15] Van Emden H.F. (1966). Studies on the relations of insect and host plant III. A comparison of the reproduction of *Brevicoryne brassicae* and *Myzus persicae* (Hemiptera: Aphididae) on Brussels sprout plants supplied with different rates of nitrogen and potassium. Entomol. Exp. Appl..

[16] Van Emden H.F., Bashford M.A. (1969). A comparison of the reproduction of *Brevicoryne brassicae* and *Myzus persicae* in relation to soluble nitrogen concentration and leaf age (leaf position) in the Brussels Sprout plant. Entomol. Exp. Appl..

[17] Merritt S.Z. (1996). Within-plant variation in concentrations of amino acids, sugar, and sinigrin in phloem sap of black mustard, *Brassica nigra* (L.) Koch (Cruciferae). J. Chem. Ecol..

[18] Jakobs R., Schweiger R., Müller C. (2019). Aphid infestation leads to plant part-specific changes in phloem sap chemistry, which may indicate niche construction. New Phytol..

[19] Johnson M.T.J. (2008). Bottom-up effects of plant genotype on aphids, ants, and predators. Ecology.

[20] Pringle E.G., Novo A., Ableson I., Barbehenn R.V., Vannette R.L. (2014). Plant-derived differences in the composition of aphid honeydew and their effects on colonies of aphid-tending ants. Ecol. Evol..

[21] Züst T., Agrawal A.A. (2017). Plant chemical defense indirectly mediates aphid performance via interactions with tending ants. Ecology.

[22] Banks C.J. (1958). Effects of the ant *Lasius niger* (L.), on the behaviour and reproduction of the black bean aphid, *Aphis fabae* Scop. Bull. Entomol. Res..

[23] Collins C.M., Leather S.R. (2002). Ant-mediated dispersal of the black willow aphid *Pterocomma salicis* L.; does the ant *Lasius niger* L. judge aphid-host quality?. Ecol. Entomol..

[24] Reilly J.J., Sterling W.L. (1983). Interspecific association and dispersion patterns of the red imported fire ant, aphids and some predaceous insects in a cotton agroecosystem. Environ. Entomol..

[25] Ascunce M.S., Yang C.-C., Oakey J., Calcaterra L., Wu W.-J., Shih C.-J., Goudet J., Ross K.G., Shoemaker D. (2011). Global invasion history of the fire ant *Solenopsis invicta*. Science.

[26] Kaplan I., Eubanks M.D. (2002). Disruption of cotton aphid (Homoptera: Aphididae)—Natural enemy dynamics by red imported fire ants (Hymenoptera: Formicidae). Environ. Entomol..

[27] Diaz R., Knutson A., Bernal J.S. (2004). Effect of the red imported fire ant on cotton aphid population density and predation of bollworm and beet armyworm eggs. J. Econ. Entomol..

[28] Mondor E.B., Rosenheim J.A., Addicott J.F. (2005). Predator-induced transgenerational phenotypic plasticity in the cotton aphid. Oecologia.

[29] Wool D., Hales D., Sunnucks P. (1995). Host plant relationships of *Aphis gossypii* Glover (Hemiptera: Aphididae) in Australia. Aust. J. Entomol..

[30] Watt M., Hales D.F. (1996). Dwarf phenotype of the cotton aphid, *Aphis gossypii* Glover (Hemiptera: Aphididae). Aust. J. Entomol..

[31] Abisgold J., Simpson S.J., Douglas A.E. (1994). Nutrient regulation in the pea aphid *Acyrthosiphon pisum*—Application of a novel geometric framework to sugar and amino acid consumption. Physiol. Entomol..

[32] Karley A.J., Douglas A.E., Parker W.E. (2002). Amino acid composition and nutritional quality of potato leaf phloem sap for aphids. J. Exp. Biol..

[33] Pervez A., Yadav M. (2018). Foraging behaviour of predaceous ladybird beetles: A review. Eur. J. Environ. Sci..

[34] Humphreys R.K., Ruxton G.D., Karley A.J. (2021). Post-dropping behavior of potato aphids (*Macrosiphum euphorbiae*). J. Insect Behav..

[35] Stephens D.W., Krebs J.R.B. (1986). Foraging Theory.

[36] Harmon J.P., Losey J.E., Ives A.R. (1998). The role of vision and color in the close proximity foraging behavior of four coccinellid species. Oecologia.

[37] Leigh Jr E.G. (2010). The evolution of mutualism. J. Evol. Biol..

[38] Xu T., Chen L. (2021). Chemical communication in ant-hemipteran mutualism: Potential implications for ant invasions. Curr. Opin. Insect Sci..

[39] Helms K., Vinson S. (2002). Widespread association of the invasive ant *Solenopsis invicta* with an invasive mealybug. Ecology.

[40] Guimarães Donatti-Ricalde M., de Carvalho Silva A., Perrone Ricalde M., Ribeiro Costa Rouws J., José MayhÉ-Nunes A., Carlos de Souza Abboud A. (2023). Abundance of natural enemies and aphids in okra crops (*Abelmoschus esculentus*—Malvaceae) diversified with *Tithonia rotundifolia* (Asteraceae). Biol. Control.

[41] Wilder S.M., Barnum T.R., Holway D.A., Suarez A.V., Eubanks M.D. (2013). Introduced fire ants can exclude native ants from critical mutualist-provided resources. Oecologia.

[42] Wilder S.M., Holway D.A., Suarez A.V., LeBrun E.G., Eubanks M.D. (2011). Intercontinental differences in resource use reveal the importance of mutualisms in fire ant invasions. Proc. Natl. Acad. Sci. USA.

[43] Rice K.B., Eubanks M.D. (2013). No enemies needed: Cotton aphids (Hemiptera: Aphididae) directly benefit from red imported fire ant (Hymenoptera: Formicidae) tending. Fla. Entomol..

[44] Shingleton A.W., Stern D.L., Foster W.A. (2005). The origin of a mutualism: A morphological trait promoting the evolution of ant-aphid mutualisms. Evolution.

[45] Xu T., Xu M., Lu Y., Zhang W., Sun J., Zeng R., Turlings T.C.J., Chen L. (2021). A trail pheromone mediates the mutualism between ants and aphids. Curr. Biol..

[46] Stadler B., Dixon A.F.G. (1998). Costs of ant attendance for aphids. J. Anim. Ecol..

[47] Yao I., Shibao H., Akimoto S. (2000). Costs and benefits of ant attendance to the drepanosiphid aphid *Tuberculatus quercicola*. Oikos.

[48] Katayama N., Suzuki N. (2002). Cost and benefit of ant attendance for *Aphis craccivora* (Hemiptera: Aphididae) with reference to aphid colony size. Can. Entomol..

[49] Vantaux A., Schillewaert S., Parmentier T., Van den Ende K., Billen J., Wenseleers T. (2015). The cost of ant attendance and melezitose secretion in the black bean aphid *Aphis fabae*. Ecol. Entomol..

[50] Nelson A.S., Mooney K.A. (2021). Comparing the individual and combined effects of ant attendance and wing formation on aphid body size and reproduction. Ann. Entomol. Soc. Am..

