# Supplementary material for: Contrasting Effects of Mutualistic Ants (Solenopsis invicta) and Predatory Ladybugs on the Proportion of Dark Green Morphs of Cotton Aphids
Source: Insects. 2025 Mar 4;16(3):271. doi: 10.3390/insects16030271 (PMC11943279; doi:10.3390/insects16030271)
Supplement: Supplementary file 1 [file insects-16-00271-s001.zip › insects-3482517-supplementary.pdf]

The script of Generalized linear model used for analyzing the proportions of dark green morphs on a seedling, on days 11, 13, 15, 17.

```
* Generalized linear model.
GENLIN The proportion of dark green aphids BY Ant Day (ORDER=ASCENDING)
  /MODEL Ant Day Ant*Day INTERCEPT=YES
 DISTRIBUTION=NORMAL LINK=IDENTITY
  /CRITERIA SCALE=MLE COVB=MODEL PCONVERGE=1E-006(ABSOLUTE) SINGULAR=1E-012
ANALYSISTYPE=3(WALD)
    CILEVEL=95 CITYPE=WALD LIKELIHOOD=FULL
  /EMMEANS TABLES=Ant SCALE=ORIGINAL COMPARE=Ant CONTRAST=PAIRWISE
PADJUST=LSD
  /EMMEANS TABLES=Day SCALE=ORIGINAL COMPARE=Day CONTRAST=PAIRWISE
PADJUST=LSD
  /EMMEANS TABLES=Ant*Day SCALE=ORIGINAL COMPARE=Ant*Day CONTRAST=PAIRWISE
PADJUST=LSD
  /MISSING CLASSMISSING=EXCLUDE
  /PRINT CPS DESCRIPTIVES MODELINFO FIT SUMMARY SOLUTION.
```

The script of Generalized linear model used for analyzing the proportions of dark green morphs among the total number of aphids that distributed on different parts of seedling.

```
* Generalized linear model.
GENLIN The proportion of dark green aphids BY Ant Plant part Day (ORDER=ASCENDING)
  /MODEL Ant Plant part Day Ant*Plant part Plant part*Day Ant*Day Ant*Plant part*Day
INTERCEPT=YES
 DISTRIBUTION=NORMAL LINK=IDENTITY
  /CRITERIA SCALE=MLE COVB=MODEL PCONVERGE=1E-006(ABSOLUTE) SINGULAR=1E-012
ANALYSISTYPE=3(WALD)
    CILEVEL=95 CITYPE=WALD LIKELIHOOD=FULL
  /EMMEANS TABLES=Ant SCALE=ORIGINAL COMPARE=Ant CONTRAST=PAIRWISE
PADJUST=LSD
  /EMMEANS TABLES=Plant part SCALE=ORIGINAL COMPARE=Plant part CONTRAST=PAIRWISE
PADJUST=LSD
  /EMMEANS TABLES=Day SCALE=ORIGINAL COMPARE=Day CONTRAST=PAIRWISE
PADJUST=LSD
  /EMMEANS TABLES=Ant*Plant part SCALE=ORIGINAL COMPARE=Ant*Plant part
CONTRAST=PAIRWISE PADJUST=LSD
  /EMMEANS TABLES=Plant part*Day SCALE=ORIGINAL COMPARE=Plant part*Day
CONTRAST=PAIRWISE PADJUST=LSD
  /EMMEANS TABLES=Ant*Day SCALE=ORIGINAL COMPARE=Ant*Day CONTRAST=PAIRWISE
PADJUST=LSD
  /EMMEANS TABLES=Ant*Plant part*Day SCALE=ORIGINAL COMPARE=Ant*Plant part*Day
CONTRAST=PAIRWISE PADJUST=LSD
  /MISSING CLASSMISSING=EXCLUDE
  /PRINT CPS DESCRIPTIVES MODELINFO FIT SUMMARY SOLUTION
  /SAVE MEANPRED CIMEANPREDL CIMEANPREDU XBPRED XBSTDERROR COOK LEVERAGE
RESID PEARSONRESID
    STDPEARSONRESID DEVIANCERESID STDDEVIANCERESID LIKELIHOODRESID.
```

The script of Generalized linear model used for analyzing the body length of dark green adults and yellow adults distributed on leaf and SPSs (stems, petioles and sprouts).

```
* Generalized linear model.
GENLIN Body length BY Ant Plant part Body color (ORDER=ASCENDING)
  /MODEL Plant part Ant Body color Ant*Plant part Plant part*Body color Ant*Body color Ant*Plant part*Body color INTERCEPT=YES
 DISTRIBUTION=NORMAL LINK=IDENTITY
  /CRITERIA SCALE=MLE COVB=MODEL PCONVERGE=1E-006(ABSOLUTE) SINGULAR=1E-012 ANALYSISTYPE=3(WALD)
    CILEVEL=95 CITYPE=WALD LIKELIHOOD=FULL
  /EMMEANS TABLES=Plant part SCALE=ORIGINAL COMPARE=Plant part CONTRAST=PAIRWISE PADJUST=LSD
  /EMMEANS TABLES=Ant SCALE=ORIGINAL COMPARE=Ant CONTRAST=PAIRWISE PADJUST=LSD
  /EMMEANS TABLES=Body color SCALE=ORIGINAL COMPARE=Body color CONTRAST=PAIRWISE PADJUST=LSD
  /EMMEANS TABLES=Ant*Plant part SCALE=ORIGINAL COMPARE=Ant*Plant part CONTRAST=PAIRWISE PADJUST=LSD
  /EMMEANS TABLES=Plant part*Body color SCALE=ORIGINAL COMPARE=Plant part*Body color CONTRAST=PAIRWISE PADJUST=LSD
  /EMMEANS TABLES=Ant*Body color SCALE=ORIGINAL COMPARE=Ant*Body color CONTRAST=PAIRWISE PADJUST=LSD
  /EMMEANS TABLES=Ant*Plant part*Body color SCALE=ORIGINAL COMPARE=Ant*Plant part*Body color CONTRAST=PAIRWISE PADJUST=LSD
  /MISSING CLASSMISSING=EXCLUDE
  /PRINT CPS DESCRIPTIVES MODELINFO FIT SUMMARY SOLUTION
  /SAVE MEANPRED CIMEANPREDL CIMEANPREDU XBPRED XBSTDERROR COOK LEVERAGE RESID PEARSONRESID
    STDPEARSONRESID DEVIANCERESID STDDEVIANCERESID LIKELIHOODRESID.
```

The script of Generalized linear model used for analyzing the head width of dark green adults and yellow adults distributed on leaf and SPSs (stems, petioles and sprouts).

```
* Generalized linear model.
GENLIN Head width BY Ant Plant part Body color (ORDER=ASCENDING)
  /MODEL Plant part Ant Body color Ant*Plant part Plant part*Body color Ant*Body color Ant*Plant part*Body color INTERCEPT=YES
 DISTRIBUTION=NORMAL LINK=IDENTITY
  /CRITERIA SCALE=MLE COVB=MODEL PCONVERGE=1E-006(ABSOLUTE) SINGULAR=1E-012 ANALYSISTYPE=3(WALD)
    CILEVEL=95 CITYPE=WALD LIKELIHOOD=FULL
  /EMMEANS TABLES=Plant part SCALE=ORIGINAL COMPARE=Plant part CONTRAST=PAIRWISE PADJUST=LSD
  /EMMEANS TABLES=Ant SCALE=ORIGINAL COMPARE=Ant CONTRAST=PAIRWISE PADJUST=LSD
  /EMMEANS TABLES=Body color SCALE=ORIGINAL COMPARE=Body color CONTRAST=PAIRWISE PADJUST=LSD
  /EMMEANS TABLES=Ant*Plant part SCALE=ORIGINAL COMPARE=Ant*Plant part CONTRAST=PAIRWISE PADJUST=LSD
  /EMMEANS TABLES=Plant part*Body color SCALE=ORIGINAL COMPARE=Plant part*Body color CONTRAST=PAIRWISE PADJUST=LSD
  /EMMEANS TABLES=Ant*Body color SCALE=ORIGINAL COMPARE=Ant*Body color CONTRAST=PAIRWISE PADJUST=LSD
  /EMMEANS TABLES=Ant*Plant part*Body color SCALE=ORIGINAL COMPARE=Ant*Plant part*Body color CONTRAST=PAIRWISE PADJUST=LSD
  /MISSING CLASSMISSING=EXCLUDE
  /PRINT CPS DESCRIPTIVES MODELINFO FIT SUMMARY SOLUTION
  /SAVE MEANPRED CIMEANPREDL CIMEANPREDU XBPRED XBSTDERROR COOK LEVERAGE RESID PEARSONRESID
    STDPEARSONRESID DEVIANCERESID STDDEVIANCERESID LIKELIHOODRESID.
```

The script of Generalized linear model used for analyzing the proportions of dark green aphids on the seedling on days 0, 2, 4 and 6, in the presence or absence of a ladybug.

```
* Generalized linear model.
GENLIN The proportions of dark green aphids BY LadybugDay (ORDER=ASCENDING)
  /MODEL Ladybug Day Ladybug*Day INTERCEPT=YES
 DISTRIBUTION=NORMAL LINK=IDENTITY
  /CRITERIA SCALE=MLE COVB=MODEL PCONVERGE=1E-006(ABSOLUTE) SINGULAR=1E-012
ANALYSISTYPE=3(WALD)
    CILEVEL=95 CITYPE=WALD LIKELIHOOD=FULL
  /EMMEANS TABLES=Ladybug SCALE=ORIGINAL COMPARE=Ladybug CONTRAST=PAIRWISE
PADJUST=LSD
  /EMMEANS TABLES=Day SCALE=ORIGINAL COMPARE=Day CONTRAST=PAIRWISE
PADJUST=LSD
  /EMMEANS TABLES=Ladybug*Day SCALE=ORIGINAL COMPARE=Ladybug*Day
CONTRAST=PAIRWISE PADJUST=LSD
  /MISSING CLASSMISSING=EXCLUDE
  /PRINT CPS DESCRIPTIVES MODELINFO FIT SUMMARY SOLUTION
  /SAVE MEANPRED CIMEANPREDL CIMEANPREDU XBPRED XBSTDERROR COOK LEVERAGE
RESID PEARSONRESID
    STDPEARSONRESID DEVIANCERESID STDDEVIANCERESID LIKELIHOODRESID.
```

The script of Generalized linear model used for analyzing the proportions of dark green aphids on leaves or SPSs of cotton seedlings, in the presence or absence of a ladybug.

```
* Generalized linear model.
GENLIN The proportions of dark green aphids BY Ladybug Plant part Day (ORDER=ASCENDING)
  /MODEL Ladybug Plant part Day Ladybug*Plant part Plant part*Day Ladybug*Day Ladybug*Plant part*Day INTERCEPT=YES
 DISTRIBUTION=NORMAL LINK=IDENTITY
  /CRITERIA SCALE=MLE COVB=MODEL PCONVERGE=1E-006(ABSOLUTE) SINGULAR=1E-012 ANALYSISTYPE=3(WALD)
    CILEVEL=95 CITYPE=WALD LIKELIHOOD=FULL
  /EMMEANS TABLES=Ladybug SCALE=ORIGINAL COMPARE=Ladybug CONTRAST=PAIRWISE PADJUST=LSD
  /EMMEANS TABLES=Plant part SCALE=ORIGINAL COMPARE=Plant part CONTRAST=PAIRWISE PADJUST=LSD
  /EMMEANS TABLES=Day SCALE=ORIGINAL COMPARE=Day CONTRAST=PAIRWISE PADJUST=LSD
  /EMMEANS TABLES=Ladybug*Plant part SCALE=ORIGINAL COMPARE=Ladybug*Plant part CONTRAST=PAIRWISE PADJUST=LSD
  /EMMEANS TABLES=Plant part*Day SCALE=ORIGINAL COMPARE=Plant part*Day CONTRAST=PAIRWISE PADJUST=LSD
  /EMMEANS TABLES=Ladybug*Day SCALE=ORIGINAL COMPARE=Ladybug*Day CONTRAST=PAIRWISE PADJUST=LSD
  /EMMEANS TABLES=Ladybug*Plant part*Day SCALE=ORIGINAL COMPARE=Ladybug*Plant part*Day CONTRAST=PAIRWISE PADJUST=LSD
  /MISSING CLASSMISSING=EXCLUDE
  /PRINT CPS DESCRIPTIVES MODELINFO FIT SUMMARY SOLUTION
  /SAVE MEANPRED CIMEANPREDL CIMEANPREDU XBPRED XBSTDERROR COOK LEVERAGE RESID PEARSONRESID
    STDPEARSONRESID DEVIANCERESID STDDEVIANCERESID LIKELIHOODRESID.
```

The script of Generalized linear model used for analyzing the difference between the numbers of aphids distributed on different parts of seedlings, in the presence or absence of predatory ladybug on Day 0, 2, 4, 6.

```
* Generalized linear model.
GENLIN The proportions of aphids BY Ladybug Plant part Day (ORDER=ASCENDING)
  /MODEL Ladybug Plant part Day Ladybug*Plant part Plant part*Day Ladybug*Day Ladybug*Plant part*Day
INTERCEPT=YES
 DISTRIBUTION=NORMAL LINK=IDENTITY
  /CRITERIA SCALE=MLE COVB=MODEL PCONVERGE=1E-006(ABSOLUTE) SINGULAR=1E-012
ANALYSISTYPE=3(WALD)
    CILEVEL=95 CITYPE=WALD LIKELIHOOD=FULL
  /EMMEANS TABLES=Ladybug SCALE=ORIGINAL COMPARE=Ladybug CONTRAST=PAIRWISE
PADJUST=LSD
  /EMMEANS TABLES=Plant part SCALE=ORIGINAL COMPARE=Plant part CONTRAST=PAIRWISE
PADJUST=LSD
  /EMMEANS TABLES=Day SCALE=ORIGINAL COMPARE=Day CONTRAST=PAIRWISE
PADJUST=LSD
  /EMMEANS TABLES=Ladybug*Plant part SCALE=ORIGINAL COMPARE=Ladybug*Plant part
CONTRAST=PAIRWISE PADJUST=LSD
  /EMMEANS TABLES=Plant part*Day SCALE=ORIGINAL COMPARE=Plant part*Day
CONTRAST=PAIRWISE PADJUST=LSD
  /EMMEANS TABLES=Ladybug*Day SCALE=ORIGINAL COMPARE=Ladybug*Day
CONTRAST=PAIRWISE PADJUST=LSD
  /EMMEANS TABLES=Ladybug*Plant part*Day SCALE=ORIGINAL COMPARE=Ladybug*Plant part*Day
CONTRAST=PAIRWISE PADJUST=LSD
  /MISSING CLASSMISSING=EXCLUDE
  /PRINT CPS DESCRIPTIVES MODELINFO FIT SUMMARY SOLUTION
  /SAVE MEANPRED CIMEANPREDL CIMEANPREDU XBPRED XBSTDERROR COOK LEVERAGE
RESID PEARSONRESID
    STDPEARSONRESID DEVIANCERESID STDDEVIANCERESID LIKELIHOODRESID.
```
